# Epidemiological and Clinical Baseline Characteristics as Predictive Biomarkers of Response to Anti-VEGF Treatment in Patients with Neovascular AMD

**DOI:** 10.1155/2016/4367631

**Published:** 2016-03-17

**Authors:** Miltiadis K. Tsilimbaris, Maria I. López-Gálvez, Roberto Gallego-Pinazo, Philippe Margaron, George N. Lambrou

**Affiliations:** ^1^Department of Ophthalmology, University of Crete Medical School, Heraklion, 70013 Crete, Greece; ^2^Department of Ophthalmology, HCU and IOBA, University of Valladolid, Valladolid, Spain; ^3^Unit of Macula, Department of Ophthalmology, University and Polytechnic Hospital La Fe, Valencia, Spain; ^4^Novartis Pharma AG, Basel, Switzerland; ^5^Vision Institute, National Center of Ophthalmology, Paris, France

## Abstract

*Purpose*. To review the current literature investigating patient response to antivascular endothelial growth factor-A (VEGF) therapy in the treatment of neovascular age-related macular degeneration (nAMD) and to identify baseline characteristics that might predict response.* Method*. A literature search of the PubMed database was performed, using the keywords: AMD, anti-VEGF, biomarker, optical coherence tomography, treatment outcome, and predictor. The search was limited to articles published from 2006 to date. Exclusion criteria included phase 1 trials, case reports, studies focusing on indications other than nAMD, and oncology.* Results*. A total of 1467 articles were identified, of which 845 were excluded. Of the 622 remaining references, 47 met all the search criteria and were included in this review.* Conclusion*. Several baseline characteristics correlated with anti-VEGF treatment response, including best-corrected visual acuity, age, lesion size, and retinal thickness. The majority of factors were associated with disease duration, suggesting that longer disease duration before treatment results in worse treatment outcomes. This highlights the need for early treatment for patients with nAMD to gain optimal treatment outcomes. Many of the identified baseline characteristics are interconnected and cannot be evaluated in isolation; therefore multivariate analyses will be required to determine any specific relationship with treatment response.

## 1. Introduction

Age-related macular degeneration (AMD) is the leading cause of blindness in the aging population of industrialized societies [1, 2], responsible for 50% of cases [3]. Neovascular AMD (nAMD), while representing only 10–20% of AMD cases, has been reported to be responsible for 80–90% of severe vision loss and/or legal blindness in this population [4, 5].

Vascular endothelial growth factors (VEGF) constitute a family of related molecules with proangiogenic properties (VEGF-A, VEGF-B, VEGF-C, VEGF-D, VEGF-E, and placental growth factor) [6, 7]. Uncontrolled expression of VEGF results in growth of new blood vessels that develop abnormalities and fail to mature [6]. This can cause vascular fragility, exudation, and bleeding, as occurs during nAMD [6]. The current standard of care for nAMD is the intravitreal administration of anti-VEGF-A drugs [8]. Three anti-VEGF therapies that target VEGF-A have been approved for intraocular use in nAMD cases: (1) ranibizumab (Lucentis®; Roche Ltd., Basel, Switzerland; Novartis Pharma AG, Basel, Switzerland) [9], (2) aflibercept (Eylea®; Regeneron Pharmaceuticals, NY, USA; Bayer Pharma AG, Berlin, Germany) [10], and (3) pegaptanib (Macugen®; OSI Pharmaceuticals, NY, USA; Pfizer, NY, USA) [11]. Bevacizumab (Avastin®; Roche Ltd., Basel, Switzerland) is licensed for use in colorectal cancer but is used off-label to treat nAMD [8, 12].

Variation in patient responses to anti-VEGF therapy has been seen in clinical trials. In the MARINA and ANCHOR studies of ranibizumab for the treatment of nAMD, the mean change from baseline in visual acuity (VA) at 24 months for the ranibizumab 0.5 mg group was +6.6 letters and +10.7 letters, respectively [13, 14]. The majority of patients treated with ranibizumab 0.5 mg achieved improvements or maintained VA at month 24 in both studies, with over 30% of patients achieving a 15-letter improvement or more from baseline [13, 14]. However, a small subset of around 10% of patients lost 15 letters or more from baseline [13, 14]. This variation in response was also seen in the HARBOR study, where 34.5% of patients who received monthly ranibizumab 0.5 mg and 33.1% of patients who received ranibizumab 0.5 mg* pro re nata* (PRN) gained 15 letters or more at month 24 from baseline [15]. Similar to findings from ANCHOR and MARINA, a small proportion of patients lost 15 letters or more from baseline with both regimens (monthly, 5.8%; PRN, 9.1%) [15].

Variation in response to anti-VEGF therapy can also be seen from differences in the frequency of injections required. In the HARBOR study, the median number of injections received by patients in the ranibizumab 0.5 mg PRN treatment arm who completed the study was 14.0; however, the range in injection frequency during the 2-year study period was from 3 to 24 injections [15].

Taken together, these data demonstrate that there is variability in patient response to therapy with anti-VEGF agents. Understanding the reasons for this variation could lead to the development of methods to predict individual patient requirements and prevent over- or undertreatment. Attendance at the eye clinic in order to receive intravitreal injections may be inconvenient and expensive for the patient, family, and caregivers; therefore, identifying the optimal injection frequency required without unnecessary clinic visits would be of benefit not only to the patient, but also to the clinic and health system. Measuring factors that could predict patient response to therapy would allow optimization of individualized patient treatment regimens, including frequency and number of injections required, thus reducing the small but real risk of injection-related adverse events, as well as improving disease management and reducing unnecessary monitoring visits.

*Aim of the Review*. In this paper, we review the current literature investigating patient response to anti-VEGF therapy in the treatment of nAMD and its subtypes and identify clinical baseline characteristics that have been found to predict patient response to anti-VEGF therapy.

## 2. Study Design

**Figure 1:**
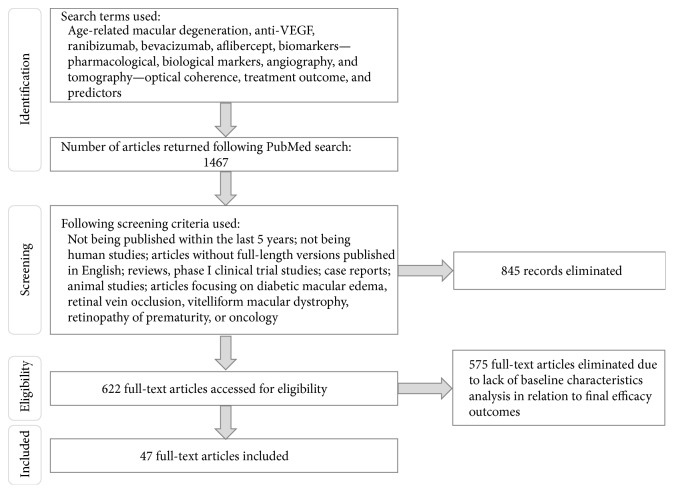
Literature search process.

A comprehensive search of the literature was conducted using the online biomedical search engine, PubMed. Search terms included age-related macular degeneration; anti-VEGF; ranibizumab; bevacizumab; aflibercept; biomarkers, pharmacological, biological markers, angiography; and tomography, optical coherence, treatment outcome, and predictors. Articles and studies were excluded if they met any of the following criteria: articles without full-length versions published in English, reviews, phase 1 clinical trial studies, case reports, and animal studies. Articles focusing on diabetic macular edema, retinal vein occlusion, vitelliform macular dystrophy, retinopathy of prematurity, or oncology also were excluded. The search was performed on June 29, 2015, and all articles meeting the search criteria, from January 2006 onward, were included in this review (Figure 1). The included articles were grouped and reviewed by overall study type (prospective phase 3, prospective nonphase 3, and retrospective studies) with the greatest review weighting given to prospective phase 3 studies (Table 1).

## 3. Baseline Factors That Predict Functional and Anatomical Responses to Treatment

The relationships between a number of baseline characteristics and treatment responses were investigated within the published literature. These broadly fell into the following groups of factors: epidemiological, functional, and anatomical/morphological.

### 3.1. Epidemiological Predictive Markers

#### 3.1.1. Age at Baseline

Retrospective subgroup analysis of the phase 3 MARINA study, which compared ranibizumab and sham treatment in patients with nAMD, used multivariate models to identify age at baseline as a statistically significant predictor of VA outcome at month 24, with increasing age associated with reduced VA gains in both treatment arms [16]. Higher age at baseline was also identified as a statistically significant predictor of worse VA outcome from a similar retrospective subgroup analysis of first-year results from the phase 3 ANCHOR study, which compared ranibizumab and verteporfin photodynamic therapy (vPDT) in patients with nAMD [17]. Patients receiving ranibizumab during MARINA and ANCHOR entered an open-label extension study, HORIZON, and subsequent long-term follow-up analysis (7-8 years after initial study enrollment) was performed in the SEVEN-UP study [18]. Comparisons of patient age and final letter score measured during the SEVEN-UP study confirmed that older patients had significantly poorer visual outcomes in this patient population (*p* = 0.027) [18]. A small (*N* = 31) prospective, single-arm, 24-month study using ranibizumab for the treatment of retinal angiomatous proliferation (RAP) also identified a negative correlation between age at baseline and final best-corrected VA (BCVA; *R* = −0.357, *p* = 0.049, Spearman's rho test), although when analyzed using multiple linear regression analysis it narrowly missed significance (*p* = 0.051) [19].

Multivariate analysis of a cohort study within the phase 3 CATT trial of bevacizumab versus ranibizumab in patients with nAMD identified older age at baseline as a predictor of worse VA score at year 1 (*p* = 0.0006) and less overall VA gain (*p* = 0.003) in both treatment arms [20].

Retrospective analysis of medical records from patients with nAMD treated with ranibizumab supports these phase 3 data, with a Pearson correlation test identifying increasing age as significantly associated with a worse visual prognosis at month 12 for patients with nAMD (*p* = 0.02), but interestingly not those with polypoidal choroidal vasculopathy (PCV; *p* > 0.22) [21]. However, a separate retrospective interventional cohort study of ranibizumab treatment showed an association between age and response at month 24 in patients with PCV (*p* = 0.03), but not those with nAMD (*p* = 0.87) using univariate logistic analyses [22]. Age at baseline was also identified as a predictor of VA response at 3 to 12 months in retrospective analyses of patients receiving bevacizumab [23–25] and ranibizumab [26].

#### 3.1.2. Duration of Disease and Previous Treatment

Although not supported by long-term data from large prospective studies, a shorter duration of disease prior to initiating anti-VEGF treatment was associated with better VA outcomes at 6 months in two retrospective studies [23, 27].

Similarly, two 6-month retrospective studies identified an association between treatment status at baseline and final outcomes, with treatment-naïve patients achieving a greater reduction in central retinal thickness (CRT) [28] and better VA [24] compared with those who had received prior treatment for nAMD. However, the treatment-naïve status of patients could also be associated with shorter disease duration, but details on disease duration were not recorded and so the actual relevance of treatment status is difficult to determine. Unfortunately there are no data from phase 3 studies to support these findings either way.

### 3.2. Functional Predictive Markers

#### 3.2.1. Best-Corrected Visual Acuity

Multivariate analysis of the MARINA study identified VA at baseline as a significant predictor of VA outcome at month 24, with higher VA at baseline associated with a smaller gain from baseline in VA at month 24 [16]. Similarly, multivariate analysis of baseline VA score for patients in the ANCHOR study showed a high correlation with the change in VA score at month 12 compared with baseline; a higher baseline VA resulted in less gain in VA from baseline at month 12, but a higher overall VA score at month 12 [17]. Indeed, baseline VA was the most influential predictor of VA outcomes at month 12 identified by this analysis [17]. Pooled data from the ranibizumab treatment arms of MARINA, ANCHOR, PIER, and SAILOR were analyzed to identify early (≥15-letter gain at month 3 from baseline) and delayed (≥15-letter gain at month 12 from baseline) responders [29]. Comparison of baseline characteristics of these two groups using Student's *t*-test showed that early responders had statistically significant lower mean baseline VA compared with delayed responders (*p* < 0.05) in the MARINA and ANCHOR trials, where patients received ranibizumab monthly, but not in the PIER and SAILOR trials, where patients received ranibizumab quarterly and PRN, respectively [29]. No other statistically significant differences in baseline characteristics were identified between the groups [29].

The CATT subanalysis performed by Ying et al. identified worse baseline VA as a statistically significant predictor of worse VA score at year 1 (*p* < 0.0001) and a baseline VA of ≥20/40 predictive of less VA gain at year 1 (*p* < 0.0001), irrespective of treatment arm [20]. A separate prospective cohort study of CATT also identified an association between baseline VA and an increased risk of outer retinal tubulations at week 104 (*p* = 0.003), irrespective of treatment arm [30]. Eyes with outer retinal tubulations at week 104 had worse VA compared with those without [30].

Post hoc analysis of the prospective, phase 3 VIEW study in patients with nAMD receiving ranibizumab or aflibercept showed a robust influence of baseline BCVA on visual outcomes at week 52 regardless of treatment arm, using a multivariate linear regression model (*p* ≤ 0.0001) [33].

Two small (*N* = 31 and *N* = 34) prospective studies of ranibizumab use in patients with nAMD also identified an association between baseline BCVA and final VA outcome at month 12 [38] and month 24 [19].

Retrospective review of medical records supports these phase 3 data and baseline VA was identified as an important predictor of VA outcomes for patients with nAMD receiving bevacizumab for 6 months [23, 24, 28, 42] and 12 months [43] and for patients receiving ranibizumab for 3 to 6 months [4, 26, 48, 51, 56], 12 months [21, 41, 49, 54, 55, 57, 60], and 3 to 4 years [45].

### 3.3. Anatomical and Morphological Predictive Markers

#### 3.3.1. Lesion Characteristics

Retrospective analysis of MARINA showed that increased lesion size at baseline was negatively associated with VA outcomes at month 24 (*p* < 0.0001) in response to ranibizumab treatment; for every increase in baseline choroidal neovascularization (CNV) lesion size of 3.6 disc areas, the improvement in VA at the study endpoint was reduced by 5 letters [16]. Subgroup analysis of ANCHOR also identified an association between lesion size and ranibizumab treatment outcome, with smaller lesion size associated with a greater gain in letters at month 12 compared with baseline [17]. Indeed, CNV lesion size was the second most influential predictor of VA outcomes at month 12, after baseline VA, identified by this analysis [17].

CNV lesion size as a predictor of response to therapy has also been observed with bevacizumab in subanalyses of CATT. Larger CNV lesion size at baseline predicted a lower overall VA score at month 12 (*p* = 0.001) and reduced gains in VA score at month 12 compared with baseline, irrespective of treatment arm (*p* = 0.02) [20]. Large lesion size was also associated with an increased risk of outer retinal tubulations at week 104 (*p* = 0.01) [30]. However, blood content of lesions at baseline did not affect final VA outcomes at years 1 and 2 (lesions composed of >50% blood versus ≤50% blood) [31]. A separate subanalysis of CATT assessed patients who developed sustained VA loss at year 2 [36]. Baseline factors independently associated with a higher incidence of VA loss included a larger area of CNV (*p* = 0.007) and presence of nonfoveal geographic atrophy (*p* = 0.006) [36]. These eyes also had more scarring (*p* = 0.007) and hemorrhage (*p* = 0.03), compared with patients without sustained vision loss [36]. Indeed, geographic atrophy at baseline was considered a significant predictor of worse VA outcome at month 12 by multivariate analysis (*p* = 0.02) [20]. Geographic atrophy was also associated with an increased risk of outer retinal tubulations at week 104 (*p* = 0.0007) [30]. Long-term follow-up of patients from MARINA and ANCHOR in the SEVEN-UP study showed that macular atrophy lesion size was the only macular anatomic variable demonstrating a significant association with final VA outcome after 7 to 8 years of treatment, using multivariate linear regression (*p* < 0.001) [18].

Lesion subtype may also predict response to anti-VEGF therapy. A retrospective study of patient medical records after treatment with bevacizumab or ranibizumab for 1 year showed that predominantly or minimally classic lesions were associated with a smaller VA gain than occult lesions (*p* = 0.0003) [20]. By contrast, RAP lesions were associated with a greater VA gain (*p* = 0.03) and an increased likelihood of gaining ≥3 lines (OR, 1.9; 95% CI, 1.2–3.1) after treatment with either bevacizumab or ranibizumab for 1 year [20].

CNV lesion size was also found to be associated with VA outcomes in 3-month [48], 6-month [27], 12-month [41, 49], and 36-month [59] retrospective studies of ranibizumab use in patients with nAMD. Interestingly, the 12-month retrospective review of medical records by Yamashiro et al. reported that larger CNV lesion size at baseline was a prognostic marker for worse VA outcome in response to treatment with ranibizumab in nAMD (*p* = 0.0021), but not in PCV (*p* = 0.93) [21]. Retrospective review of medical records identified an association between CNV lesion type 1 and nonresponse to ranibizumab according to fundus findings (increase in exudative fundus findings or CRT increase >100 *μ*m after treatment) at month 12 [58].

#### 3.3.2. Vitreomacular Interface

Post hoc analysis of the phase 2 prospective study, MONT BLANC, compared the impact of the vitreomacular interface condition on outcomes of ranibizumab monotherapy versus vPDT plus ranibizumab combination therapy in patients with nAMD [35]. Analysis of variance showed that the change in BCVA from month 3 to month 12 was not significantly different between patients with posterior vitreous detachment (PVD) or vitreomacular adhesion (VMA) in either treatment arm, although combination therapy resulted in a significant loss of potential vision gain in patients with PVD [35]. The vitreomacular interface integrity had no impact on anatomic response to treatment, as measured by change in CRT [35].

Subanalysis of the prospective phase 3 trial, EXCITE, also investigated the effect of the vitreomacular interface on treatment outcomes in patients with nAMD receiving ranibizumab [32]. Similar to the MONT BLANC analysis, no significant differences were observed in BCVA and CRT outcomes at month 12 between patients with PVD and VMA at baseline [32]. However, ranibizumab monthly dosing conferred benefit over ranibizumab quarterly dosing for patients with VMA, but not PVD [32].

Retrospective review of medical records from patients with nAMD found that VMA at baseline was associated with poor treatment outcomes in response to ranibizumab at month 12 [55] and in response to ranibizumab or bevacizumab after nearly 2 years (*p* = 0.02) [61]. Lastly, the effect of an idiopathic epiretinal membrane on both visual and anatomical outcome in response to bevacizumab has been evaluated by a retrospective medical records review and found to have no significant effect at 2 years [40].

#### 3.3.3. Retinal Tissue Thickness

CATT subanalysis showed that greater foveal thickness at baseline predicted a lower overall VA score at month 12 (*p* = 0.01), irrespective of treatment arm [20]. These findings were supported by retrospective review of medical records, which showed that thicker subretinal tissue, CRT, and foveal thickness at baseline were associated with a reduced visual response after 3 to 12 months of treatment with either bevacizumab or ranibizumab in patients with nAMD [25, 27, 28, 48, 56].

#### 3.3.4. Intraretinal Cysts and Cystoid Macular Edema

Subanalysis of the EXCITE study performed by Simader et al. aimed to identify morphologic parameters relevant for visual outcome in patients with nAMD receiving ranibizumab [34]. Correlation analyses demonstrated a significantly lower mean BCVA at month 12 for patients with intraretinal cysts (IRC) at baseline compared with patients without [34].

Post hoc analysis of the VIEW study in patients with nAMD receiving ranibizumab or aflibercept showed that IRC and pigment epithelial detachment (PED) at baseline were predictive of a negative treatment outcome at week 52 regardless of treatment arm, using a multivariate linear regression model (*p* ≤ 0.0001 and *p* = 0.028, resp.) [33]. The volume of fibrovascular PED at baseline was also associated with impaired BCVA at month 24 in a separate prospective study of patients with nAMD receiving ranibizumab (*p* = 0.011) [37].

Retrospective analyses of medical records from patients with nAMD receiving bevacizumab for 9 to 12 months demonstrated that the presence of cystoid macular edema at baseline was significantly associated with a worse visual outcome [25, 46]. Retrospective studies also supported the association between fibrovascular PED volume size and visual outcomes for patients receiving ranibizumab [58].

#### 3.3.5. Retinal Vascular Caliber

Retinal vascular caliber can be measured by imaging the fundus and is an indirect indicator of ocular blood flow [39]. Wickremasinghe et al. reported in a prospective study of 88 patients with nAMD receiving ranibizumab that retinal vascular caliber predicted visual outcomes after intravitreal ranibizumab treatment for nAMD [39]. Retinal vascular caliber was separated into central retinal arterial equivalent (CRAE) and venular equivalent (CRVE) to represent the average caliber of arterioles and venules, respectively. Although no correlation was seen between CRAE and visual outcome using multinomial logistic regression analysis, patients experiencing deterioration in VA at 12 months compared with baseline had significantly larger CRVE at baseline (243.10 *μ*m; 95% CI, 227.01–259.19) compared with patients with stable VA (214.30 *μ*m; 95% CI, 205.79–222.81) or improved VA (215.26 *μ*m; 95% CI, 204.69–225.84; *p* = 0.007) [39].

#### 3.3.6. Outer Retinal Structures

The external limiting membrane (ELM) and the photoreceptor ellipsoid zone (EZ), formerly known as inner-segment/outer-segment junction, are markers of the integrity of the photoreceptor layer [43]. Retrospective analysis of medical records from patients with nAMD receiving bevacizumab showed that pretreatment integrity (damage) of both the ELM and EZ was significantly negatively associated with visual response after a mean of 11 months (*p* = 0.039 and *p* = 0.043, resp.) using multivariate analysis [43]. Baseline abnormalities of ELM and EZ have both been shown to be negatively associated with visual outcomes at 3 to 12 months in retrospective studies of patients with nAMD receiving ranibizumab [51, 53, 56]. However, a separate retrospective study did not find any association between EZ integrity at baseline and visual outcome at month 12 in response to ranibizumab treatment [50].

Retinal pigment epithelium (RPE) tears can occur spontaneously but can also occur as a serious complication of anti-VEGF therapy that may lead to decline or loss of vision. A retrospective evaluation of patients with serous vascular or fibrovascular PED found that RPE tears only developed in patients with serous PED (14.6%) [44]. The reason for this may be that stretching forces on the weakened RPE result in a tear [52]. In a retrospective chart review of patients with nAMD who received bevacizumab, the risk of an RPE tear increased exponentially with increased height of the PED at baseline [52].

Small dense particles (SDPs) may represent proinflammatory and proangiogenic cells, such as macrophages [47, 62]. Baseline SDPs may predict therapeutic outcomes; for example, in a retrospective study of medical records from patients receiving ranibizumab for nAMD, a significant positive correlation was observed between baseline levels of SDPs and the improvement in BCVA at month 3 (Spearman's correlation coefficient = 0.359; *p* = 0.005); however, no correlation was observed between baseline SDPs and CRT [47].

## 4. Discussion

This comprehensive literature review investigated whether any baseline characteristic could be identified that would predict a patient's response to anti-VEGF treatment. Across the different clinical trials, variation in patient response to anti-VEGF treatment was observed, in terms of both functional improvement and anatomical response [63–66]. In clinical trials in which an individualized treatment regimen was used, variation was also observed in the mean number of required treatments [15, 66]. Understanding the reasons for this variation may aid in predicting individual patient requirements and so help manage the patient's expectations in terms of both treatment outcome and burden. Such optimization of the treatment interval and treatment frequency could also prevent any over- or undertreatment and reduce the number of unnecessary monitoring visits. In addition to helping reduce the administrative load on healthcare systems, this would also benefit the patients and their carers, the clinic, and health service providers.

The baseline characteristics investigated encompassed epidemiological, functional, and anatomical categories. Epidemiological factors included the age of the patients, duration of the disease, and any previous treatment received. Functional factors included baseline VA, whereas anatomical factors focused on lesion characteristics, the vitreomacular interface, retinal tissue thickness, IRC and edema, ocular blood flow, and structural changes.

Of the several characteristics examined, lower baseline VA was found to correlate with greater VA gain in a large number of studies [4, 16–21, 23, 24, 26, 28–30, 38, 41–43, 45, 48, 49, 51, 54–57, 60]. It has been speculated that this may be mainly due to the larger capacity for improvement in patients with lower VA, whereas patients with higher baseline VA reach a plateau such that VA cannot improve further [49]. A greater VA gain may not be indicative of the absolute end VA; indeed, patients with higher baseline BCVA were found to generally achieve higher VA outcomes compared with patients who had poorer baseline VA, despite the fact that these patients with low baseline VA achieved larger absolute VA gain [17, 20, 30]. This may indicate a need to reevaluate the concept of treatment response/treatment success, where not only the gain in VA but also the end VA is evaluated.

In line with the finding that poorer starting VA is predictive of poorer end VA, was the finding that longer disease duration correlated with worse treatment outcome [23, 27]. It has been postulated that this latter finding may also be due to the mechanism of the nAMD disease progression [67]. As previously mentioned, VEGF increases vascular permeability [68], which facilitates extravasation of plasma proteins and migration of new endothelial cells, resulting in neovascularization and edema development [68]. During disease progression and vessel maturation, pericytes are recruited to the endothelial cells to form a sheath and supply VEGF and other cell survival factors to the proliferating endothelial cells [67, 69]. New vessels are dependent on VEGF and are therefore sensitive to anti-VEGF treatment, until they acquire a pericyte sheath [67, 69]. As anti-VEGF therapy influences the unprotected endothelial cells, it causes a decrease in edema and initial improvement in VA [67]. Once protected by pericytes, the neovascular complex is resistant to this VEGF inhibition; this may account for the plateau that is usually observed after initial anti-VEGF treatment, irrespective of the baseline VA [67]. Consequently, if the anti-VEGF therapy is stopped, unprotected endothelial cells may again initiate proliferation in response to VEGF, and the disease progresses [67]. Disease progression can also result in subfoveal fibrosis, particularly in patients with type 2 lesions (classic and predominantly classic CNV) [70]. This fibrous tissue is resilient to anti-VEGF treatment and can cause severe vision loss [70]. Subfoveal fibrosis may already be present at baseline in advanced lesions, which may explain why some lesions do not respond as well as others. In advanced nAMD, the neurosensory retina may be irreversibly damaged and thus fluid resolution following anti-VEGF therapy may only be associated with limited functional gains, regardless of the anatomical improvement.

The recruitment of pericytes is initiated by another growth factor, platelet-derived growth factor-BB (PDGF-BB). Based on the mechanism outlined above, inhibition of PDGF-BB would result in the stripping of pericytes from vessels, rendering them sensitive to anti-VEGF inhibition [67]. Furthermore, PDGF-BB is also involved in fibrogenesis, and so its inhibition is expected to result in the reduction of fibrosis formation [71]. The efficacy and safety of an anti-PDGF-BB agent (E10030) in combination with ranibizumab have been tested in a large phase 2b clinical trial. Indeed, in comparison with ranibizumab monotherapy, dual inhibition resulted in a 62% additional benefit from baseline [72]. Further phase 3 trials are currently in progress [67].

Further to poorer BCVA and longer disease duration, several other characteristics have also been identified as being associated with poorer response. These include older age of the patient, any previous treatment, larger CNV lesion, and larger retinal tissue thickness (Table 1). It should also be noted that the presence of PCV at baseline had a negative effect on treatment outcomes in some studies [21, 22, 45], but not others [58]. Rather than indicating a specific correlation, this may be due to inherent intercorrelation of all these characteristics with longer disease duration and baseline VA. These findings highlight the absolute need for early treatment of patients with nAMD. Because longer disease duration, as well as several characteristics associated with longer duration of the disease (relatively poor baseline BCVA, older age, previous treatment, lesion size, and retinal tissue thickness), largely correlated with a relatively poorer response, earlier treatment initiation may facilitate a better treatment response. In addition, as previously mentioned, longer disease duration and so more advanced disease may also correlate with more extensive tissue and structural damage, resulting in irreversible VA loss.

### 4.1. Study Limitations

This review was exploratory in nature, so although limited clinical conclusions can be drawn from evaluation of these studies, it does generate hypotheses that may be confirmed by larger prospective trials. Treatment parameters (e.g., regimen, retreatment criteria) and lesion evaluation techniques differed across the different studies; standardization will be required to determine any specific relationships. Because baseline characteristics are interconnected, further larger scale studies and multivariate analyses will be required to definitively confirm these. Further complexity is afforded by the lack of a uniform definition for a “treatment response.” Limited evidence exists to definitively link anatomical and functional responses, the association between a decrease in CRT and improvement in VA [73, 74]. Although it is generally believed that persistent residual fluid in the retina might have long-term implications for visual outcomes and may lead to irreversible retinal damage [75, 76], it is currently unclear whether a complete absence of fluid (“dry retina”) should be considered a therapeutic goal. It may be that, similar to VA, rather than the absolute change in retinal thickness, its relation to end retinal thickness may need to be considered in the future. As previously mentioned, the change in VA may not be indicative of treatment response and end VA should be taken into account as well, when evaluating treatment success.

## 5. Conclusions

Neovascular AMD is a multifactorial disease and it is unlikely that a single characteristic will be able to define treatment response/outcome. It may be that a tool with multiple parameters could be developed to guide and optimize the treatment of nAMD and help guide patient expectations. This paper reviewed the current literature to investigate whether a patient's response to anti-VEGF therapy could be predicted based on any baseline characteristic. Although several different parameters were identified that correlated with poorer prognosis, the majority of factors were associated with disease duration (i.e., longer disease duration results in worse treatment outcomes), highlighting the clinical importance of early treatment in the natural history of nAMD. As many of the identified baseline characteristics are interconnected and cannot be evaluated in isolation, thorough multivariate analyses will be required to determine any specific relationship with treatment response.

## Additional Points

Baseline characteristics may present a useful tool in predicting patient response to anti-VEGF treatment, helping both patients and clinicians plan appropriately. Markers suggest longer disease duration is associated with worse therapy outcomes, highlighting the importance of early treatment. Extensive multivariate analyses are required to determine specific relationships with treatment response.

## Figures and Tables

**Table 1:** Summary of studies reviewed.

Article	Duration	Tx	Indication	Patient/eye number	Study findings
Phase 3 prospective studies					
Altaweel et al., 2015 [31]	CATT: post hoc analysis (24 months)	RBZ/BVZ	nAMD	1185	Percentage blood composition of lesion did not affect VA gains at 12 and 24 months.
Bhisitkul et al., 2015 [18]	SEVEN-UP: post hoc analysis (7-8 years)	RBZ	nAMD	65	Long-term vision outcomes related to patient age but not patient gender or ethnicity. Macular atrophy lesion size was associated with VA.
Boyer et al., 2007 [16]	MARINA: post hoc analysis (24 months)	RBZ	nAMD	716	Increasing age, larger CNV lesion size at baseline, and a higher baseline VA score were all associated with less gain of VA. Most important predictors of VA outcomes were BCVA, CNV lesion size, and age.
Hariprasad et al., 2012 [29]	MARINA, ANCHOR, PIER, SAILOR: post hoc analysis (12 months)	RBZ	nAMD	1824	Lower baseline VA was associated with an early response to treatment.
Kaiser et al., 2007 [17]	ANCHOR: post hoc analysis (12 months)	RBZ	nAMD	423	Lower baseline VA, smaller baseline CNV lesion size, and younger baseline age were associated with greater gain of letters at study endpoint.
Lee et al., 2014 [30]	CATT: post hoc analysis (24 months)	RBZ/BVZ	nAMD	368	Poor VA, GA, and greater lesion size at baseline were associated independently with greater risk of ORT at 104 weeks.
Mayr-Sponer et al., 2013 [32]	EXCITE: post hoc analysis (12 months)	RBZ	nAMD	252	Patients with PVD or VMA at baseline showed no significant differences in efficacy at month 12.
Schmidt-Erfurth et al., 2015 [33]	VIEW: post hoc analysis (96 weeks)	RBZ/AFL	nAMD	1202	IRC at baseline had a negative impact on BCVA. BCVA had a robust influence on VA outcome; visual gains were higher with lower BCVA.
Simader et al., 2014 [34]	EXCITE: post hoc analysis (12 months)	RBZ	nAMD	353	IRC at baseline associated with significantly less gain in BCVA during follow-up. There was no significant difference in outcome between patients with and without SRF at baseline.
Waldstein et al., 2014 [35]	MONT BLANC: post hoc analysis (12 months)	RBZ/vPDT	nAMD	237	Patients with PVD and VMA at baseline receiving ranibizumab achieved similar VA gains and reductions in CRT at month 12.
Ying et al., 2013 [20]	CATT: post hoc analysis (12 months)	RBZ/BVZ	nAMD	1105	Older age, better baseline VA, large CNV area, predominantly or minimally classic lesion, absence of RAP lesion, presence of GA, greater foveal thickness, and RPE elevation were associated with less improvement in VA at 1 year.
Ying et al., 2014 [36]	CATT: post hoc analysis (24 months)	RBZ/BVZ	nAMD	1030	Nonfoveal GA and larger area of CNV at baseline were independently associated with a higher incidence of sustained visual loss.

Prospective studies (not phase 3)					
Hoerster et al., 2014 [37]	24 months	RBZ	nAMD	75	Volume of fibrovascular PED at baseline correlated most with impaired BCVA after 24 months.
Shin and Yu, 2014 [19]	24 months	RBZ	RAP	31	Older age, larger CNV size, and poor initial BCVA were associated with poor VA outcome. Among factors associated with poor VA outcome, only the stage of RAP remained statistically significant on multiple linear regression analysis.
Weingessel et al., 2015 [38]	12 months	RBZ	nAMD	34	Better baseline BCVA was the most important predictive factor for final BCVA.
Wickremasinghe et al., 2012 [39]	12 months	RBZ	nAMD	88	Larger baseline retinal venular caliber was significantly associated with a poorer response to treatment.

Retrospective studies					
Ahlers et al., 2009 [4]	3 months	RBZ	nAMD	30	ODR from subretinal fluid correlated with BCVA at weeks 4 and 12. Strong association between baseline BCVA and visual function at subsequent visits.
Alkin et al., 2013 [40]	24 months	BVZ	nAMD	63	Patients with idiopathic epiretinal membranes at baseline showed no significant differences in efficacy at month 12 and month 24 compared to those without.
Bloch et al., 2013 [41]	12 months	RBZ	nAMD	279	Great BCVA (≥70 letters) and smaller lesion size (<4 DA) associated with better efficacy outcomes.
Byun et al., 2010 [25]	12 months	BVZ	nAMD	113	SRT thickness and CME were associated with efficacy outcomes.
Chhablani et al., 2012 [42]	6 months	BVZ	nAMD	85	Baseline BCVA was a predictive factor for the visual outcome.
Chhablani et al., 2013 [43]	11.2 months	BVZ	nAMD	50	BCVA, IS/OS junction, and ELM damage were significant predictors for treatment effect and visual improvement.
Clemens et al., 2014 [44]	No time frame given	RBZ/BVZ	nAMD	103	Serous vascularized PED at baseline was associated with RPE tears, whereas fibrovascular PED was not.
Coco et al., 2014 [45]	1.9–5.4 years	RBZ	nAMD/PCV	299	Patients with PCV had a worse final outcome. Worse initial VA associated with atrophy at the final visit.
Fang et al., 2013 [23]	6 months	BVZ	nAMD	144	Younger age, lower baseline VA, and shorter duration of disease were significantly associated with greater VA score improvements.
Fong et al., 2008 [46]	9.4 months	BVZ	nAMD	109	Large ICR (gross CME) before treatment had increased risk of worse vision.
Framme et al., 2010 [47]	3 months	RBZ	nAMD	61	Baseline amount of SDPs correlated positively with the increase in BCVA; larger number associated with better outcome with ranibizumab therapy.
Kang et al., 2014 [48]	6 months	RBZ	nAMD	40	Baseline BCVA, baseline CNV size, and subfoveal choroidal thickness were significant prognostic factors for visual outcome.
Kang and Roh, 2009 [49]	12 months	RBZ	nAMD	64	Baseline VA and CNV size influenced VA outcomes.
Kim et al., 2014 [27]	6 months	RBZ	nAMD	91	Longer duration of symptoms, greater extent of hemorrhage, and greater CFT at baseline were correlated with poor BCVA at month 6.
Kolb et al., 2012 [50]	12 months	RBZ/BVZ	nAMD	75	CRT, IS/OS integrity, and retinal fluid did not have a predictive value regarding VA outcome.
Kwon et al., 2014 [51]	3 months	RBZ	nAMD	59	Better initial VA and greater ELM length at baseline were associated with less change in VA. Initial IS/OS-D, ELM length, and particularly ELM-D can be useful predictors of the visual outcome.
Leitritz et al., 2008 [52]	No time frame given	BVZ	nAMD	393	Risk of an RPE tear correlates with the height of the PED on OCT.
Levy et al., 2009 [24]	6 months	BVZ	nAMD	65	Eyes with better VA at baseline and without previous PDT treatment achieved better final VA. Classic membrane type and lower age somewhat associated with better posttreatment VA.
Mathew et al., 2013 [53]	12 months	RBZ	nAMD	100	Intact EZ and the ELM in the subfoveal area at BL indicated final VA at month 12. Patients with ELM have VA nearly 20 letters higher than those without.
Matsumiya et al., 2015 [22]	24 months	RBZ	nAMD/PCV	59	Typical nAMD associated with greater BCVA improvement compared with PCV at BL. Age was associated with response in PCV, but not nAMD. Greater height of PED associated with VA outcome in PCV group.
Menghini et al., 2010 [54]	12 months	RBZ	nAMD	60	Baseline VA was statistically significantly lower in good responders than in bad responders.
Nomura et al., 2014 [55]	12 months	RBZ	nAMD	123	VMA at baseline associated with poor treatment outcomes. Better baseline BCVA was associated with poor visual response.
Oishi et al., 2013 [56]	7.7 months	RBZ	nAMD	76	Baseline BCVA was the most powerful predictor for VA prognosis. ELM length, IS/OS length, and foveal thickness showed weaker correlation.
Shona et al., 2011 [57]	12 months	RBZ	nAMD	77	Poor baseline VA was a predictor of maximum gain in VA. Eyes with better baseline VA had a better final VA.
Singh et al., 2009 [28]	6 months	BVZ	nAMD	73	Worse BL BCVA associated with better final VA. Thicker CRT associated with a greater reduction in CRT. Treatment-naïve patients had a greater mean CRT reduction than those who had previously received treatment.
Suzuki et al., 2014 [58]	12 months	RBZ	nAMD	141	Initial fibrovascular PED and serous PED were associated with nonresponse as judged by BCVA. Initial fibrovascular PED and type 1 CNV were associated with nonresponse, as judged by fundus findings.
Toth et al., 2015 [59]	36 months	RBZ	nAMD	420	Regression analysis identified atrophy and fibrosis as predictors of best BCVA.
Tran et al., 2011 [60]	12 months	RBZ	nAMD	59	Baseline VA is a predictor of visual gain.
Üney et al., 2014 [61]	22.3 months	RBZ/BVZ	nAMD	61	Patients with PVD at baseline were associated with a greater rate of improved or stable BCVA, compared with patients with VMA.
van Asten et al., 2014 [26]	3 months	RBZ	nAMD	391	Independent predictors for nonresponse were age and baseline VA.
Yamashiro et al., 2012 [21]	12 months	RBZ	nAMD/PCV	105	Age, VA, and size of GLD (lesion size) were significantly associated with visual prognosis in nAMD, but not PCV.

AFL = aflibercept; BCVA = best-corrected visual acuity; BL = baseline; BVZ = bevacizumab; CFT = central foveal thickness; CME = cystoid macular edema; CNV = choroidal neovascularization; CRT = central retinal thickness; DA = disc areas; ELM = external limiting membrane; EZ = ellipsoid zone; GA = geographic atrophy; GLD = greatest linear dimension; IRC = intraretinal cysts; IS = inner segment; nAMD = neovascular age-related degeneration; ODR = optical density ratio; ORT = outer retinal tubulations; OS = outer segment; PCV = polypoidal choroidal vasculopathy; PED = pigment epithelial detachment; PVD = posterior vitreous detachment; RAP = retinal angiomatous proliferation; RBZ = ranibizumab; REP = retinal pigment epithelium; SDP = small dense particle; SRF = subretinal fluid; VA = visual acuity; VMA = vitreomacular adhesion; vPDT = verteporfin photodynamic therapy.
